# Memory maintenance by PKMζ — an evolutionary perspective

**DOI:** 10.1186/1756-6606-5-31

**Published:** 2012-09-18

**Authors:** Todd Charlton Sacktor

**Affiliations:** 1 The Robert F. Furchgott Center for Neural and Behavioral Science, Departments of Physiology, Pharmacology, and Neurology, State University of New York Downstate Medical Center, 450 Clarkson Ave, Brooklyn, NY, 10705, USA

**Keywords:** PKM zeta, PKMzeta, LTP, Memory

## Abstract

Long-term memory is believed to be maintained by persistent modifications of synaptic transmission within the neural circuits that mediate behavior. Thus, long-term potentiation (LTP) is widely studied as a potential physiological basis for the persistent enhancement of synaptic strength that might sustain memory. Whereas the molecular mechanisms that initially induce LTP have been extensively characterized, the mechanisms that persistently maintain the potentiation have not. Recently, however, a candidate molecular mechanism linking the maintenance of LTP and the storage of long-term memory has been identified. The persistent activity of the autonomously active, atypical protein kinase C (aPKC) isoform, PKMζ, is both necessary and sufficient for maintaining LTP. Furthermore, blocking PKMζ activity by pharmacological or dominant negative inhibitors disrupts previously stored long-term memories in a variety of neural circuits, including spatial and trace memories in the hippocampus, aversive memories in the basolateral amygdala, appetitive memories in the nucleus accumbens, habit memory in the dorsal lateral striatum, and elementary associations, extinction, and skilled sensorimotor memories in the neocortex. During LTP and memory formation, PKMζ is synthesized *de novo* as a constitutively active kinase. This molecular mechanism for memory storage is evolutionarily conserved. PKMζ formation through new protein synthesis likely originated in early vertebrates ~500 million years ago during the Cambrian period. Other mechanisms for forming persistently active PKM from aPKC are found in invertebrates, and inhibiting this atypical PKM disrupts long-term memory in the invertebrate model systems *Drosophila melanogaster* and *Aplysia californica*. Conversely, overexpressing PKMζ enhances memory in flies and rodents. PKMζ persistently enhances synaptic strength by maintaining increased numbers of AMPA receptors at postsynaptic sites, a mechanism that might have evolved from the general function of aPKC in trafficking membrane proteins to the apical compartment of polarized cells. This mechanism of memory may have had adaptive advantages because it is both stable and reversible, as demonstrated by the downregulation of experience-dependent, long-term increases in PKMζ after extinction and reconsolidation blockade that attenuate learned behavior. Thus, PKMζ, the “working end” of LTP, is a component of an evolutionarily conserved molecular mechanism for the persistent, yet flexible storage of long-term memory.

## 

For over a century, scientists have postulated that persistent changes in the synaptic connections among neurons might maintain long-term memory [1]. Compelling experimental support for this hypothesis came from invertebrate model systems, notably *Aplysia californica*, in which changes in synaptic strength among identified neurons mediating behavior could be directly observed [2]. In the 1980s and early 1990s, further studies in molluscan and insect model systems lead to the discovery of several signaling molecules that initiate long-term changes in synaptic transmission and behavior, including the cAMP-dependent protein kinase (PKA) [2,3] and the transcription factor, cAMP response element-binding protein (CREB) [4,5], which were then shown to be crucial for memory formation in rodents and other animals [6]. Thus by 2000, much of the learning and memory field had come to believe that the molecular mechanisms of memory are evolutionarily conserved, and the keys to understanding these mechanisms were the molecules that control synaptic plasticity [7].

The form of synaptic plasticity most widely studied in mammalian systems is long-term potentiation (LTP), a persistent synaptic enhancement first characterized in detail in the hippocampus by Bliss and Lømo [8,9]. Interest in LTP grew rapidly with the discovery that the activation of the *N*-methyl-D-aspartate receptor (NMDAR) triggers both hippocampal LTP induction [10] and hippocampus-dependent spatial learning [11]. Following these seminal findings, over a hundred signaling molecules downstream of the NMDA receptor were characterized [12]. Some, such as Ca^2+^/calmodulin-dependent protein kinase II (CaMKII), initiate a transient early-LTP [13], whereas others, like mitogen-activated protein kinase (MAPK), participate in the regulation of new protein synthesis that is crucial for the transition from early- to more persistent late-LTP [14,15]. Because many of the signaling molecules important for LTP induction were also implicated within a brief time window of an hour after learning during the initial cellular consolidation of long-term memory, the case that an LTP-like mechanism might mediate the cellular basis of memory grew stronger.

But an essential mechanism for both LTP and long-term memory was missing — a mechanism maintaining the changes in synaptic strength and the learned behavior over time. Although scores of the signaling molecules that were activated during LTP were also found to be functionally important for inducing late-LTP, none had been found necessary for maintaining the potentiation once it had been established for 1–2 hours [12,16]. Because inhibitors of protein synthesis applied during this initial time window blocked the induction of both late-LTP and many forms of long-term memory [17,18], the general assumption in the field was that newly synthesized proteins were critical for the persistence of LTP and memory, most likely to serve as building blocks for new synapses. Once these new, experience-dependent synapses had been constructed, however, they could not be eliminated by any enzymatic inhibitor. Thus, in the prevailing theory, long-term memory could be prevented from forming, but could not be erased.

In 2002, however, a brain-specific, autonomously active isozyme of PKC, PKMζ, was found to be both necessary and sufficient for maintaining the late-phase of synaptic potentiation in hippocampal slices [16]. Douglas Ling, Larry Benardo, and our colleagues showed that synapses were potentiated by intracellular perfusion of PKMζ, and late-LTP was reversed by inhibiting the kinase, even when the inhibitors were applied many hours after the initial protein synthesis-dependent time window [16,19-22]. Then in 2006, André Fenton and our colleagues showed that the PKMζ inhibitor, zeta inhibitory peptide (ZIP), which effectively blocks the action of PKMζ both biochemically *in vitro* and within neurons [16,19,23], reverses LTP *in vivo* 1 day after induction and disrupts spatial memory in the rat hippocampus 1 day or even 1 month after training [22]. The following year, Yadin Dudai and our colleagues began a series of studies showing both ZIP and dominant negative mutations of PKMζ disrupt long-term memory in rat neocortex, up to 3 months after training [24-26].

Subsequently, many forms of long-term memory in a wide variety of neural circuits were shown to be maintained by the persistent activity of PKMζ. In addition to different types of spatial long-term memories [27,28], trace memories in the hippocampus [21], aversive memories in the basolateral amygdala (BLA) [27,29-32], appetitive memories in the nucleus accumbens [33-35], habit memory in the dorsal lateral striatum [36], and elementary associations [24-26,37], extinction [38], and skilled sensorimotor memories [39] in the neocortex were all disrupted by inhibiting PKMζ. Persistent experience-dependent enhancement of synaptic transmission in the hippocampus [21] and the primary visual cortex [40] were also erased by inhibiting PKMζ. Providing an underlying cellular basis for spatial memory erasure, recent work has shown that inhibiting PKMζ disrupts the stable firing patterns of hippocampal place cells exposed to a familiar environment [41]. After the drug has been eliminated, the same place cells establish new stable firing patterns in the familiar environment that have no relationship to the old patterns that had been erased. Some forms of memory were not erased by inhibiting PKMζ, including short-term memories mediated by the hippocampus [22] and neocortex [26], and certain long-term memories characterized by the habituation of behavioral responses, such as latent inhibition and attenuation of neophobia [24].

In addition to physiological memory storage, the persistence of several neurological and psychiatric disorders that had been hypothesized to be mediated, in part, by LTP-like changes in the neural circuitry mediating pain or reward was also found to be maintained by PKMζ in animal models. Thus, ZIP ameliorates chronic neuropathic pain when injected in the anterior cingulate cortex [42-44] and spinal cord [45-48], post-traumatic stress disorder in the insular cortex [49], and addiction in nucleus accumbens [33-35], BLA [38], hippocampus [50], and ventral tegmental nucleus [51]. Abnormal aggregations of PKMζ are also observed in and near neurofibrillary tangles in the brains of individuals with Alzheimer’s disease [52].

ZIP, a cell-permeable pseudosubstrate peptide inhibitor, is the most commonly used pharmacological tool to inhibit PKMζ. ZIP applied extracellularly to neurons blocks the action of PKMζ perfused into CA1 pyramidal cells in hippocampal slices [19,23], PKMζ transfected into primary cultured hippocampal neurons [53], and PKCζ introduced into sensory neurons [47]. The IC50 of the ability of ZIP to inhibit PKMζ-mediated potentiation of α-amino-3-hydroxy-5-methyl-4-isoxazolepropionic acid receptor (AMPAR) responses at synapses of CA1 pyramidal cells is nearly identical to the IC50 of its ability to reverse late-LTP at these synapses [19]. Because both full-length atypical PKC (aPKC) isoforms, PKCζ and PKCι/λ, contain the identical pseudosubstrate sequence, ZIP is also a standard reagent to inhibit the function of full-length aPKC within cells [54] and to identify intracellular aPKC substrates [55]. One paper had suggested ZIP at the doses used to inhibit PKMζ postsynaptically perfused into neurons was not effective on a PKMζ fusion protein overexpressed in cultured cells [56]. These negative results, however, were subsequently explained to be a consequence of using the standard doses of ZIP in overexpression systems that increase kinase levels between 1–2 orders of magnitude above normal [23]. At such high levels of overexpression, the exogenous “spare” kinase, analogous to spare receptors, far exceeds the endogenous kinase, and the standard doses of ZIP that inhibit PKMζ in neurons and reverse LTP maintenance would be expected to have no noticeable effect [23].

Extending beyond maintenance to expression, Karim Nader and our colleagues at McGill University showed that PKMζ sustained late-LTP and long-term memory by a common mechanism of synaptic enhancement. PKMζ potentiates synaptic transmission by modifying the trafficking of GluA2 subunit-containing AMPARs so as to increase the number of receptors at postsynaptic sites [30,57,58] (Figure 1). Nader and our colleagues showed that blockers of GluA2 endocytosis prevent the disruption of LTP maintenance and memory storage induced by ZIP, confirming that the agent effectively inhibits PKMζ’s mechanism of action both in brain slices and *in vivo*[30,34,36].

**Figure 1:**
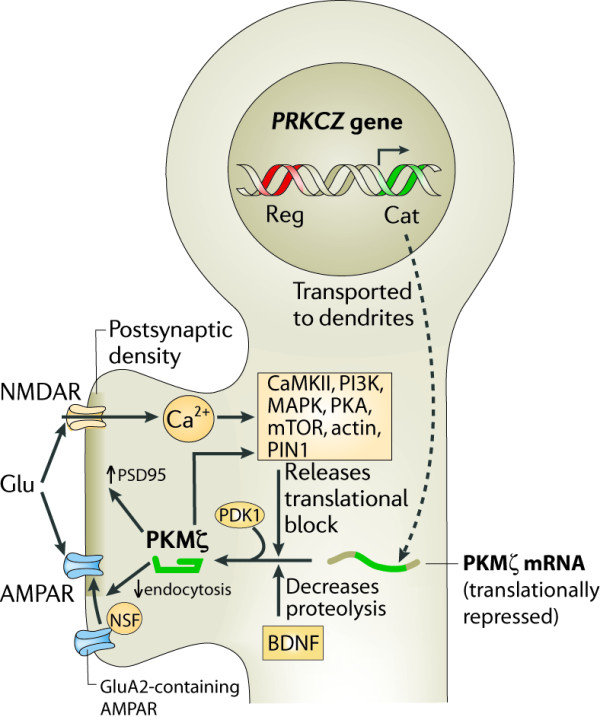
**Summary of the signaling pathways of PKMζ-mediated late-LTP.** Transcription from an internal promoter within the *PRKCZ* gene expresses a PKMζ mRNA that encodes a PKCζ catalytic domain (green) without a regulatory domain (red). The PKMζ mRNA, which is transported to dendrites of neurons, is translationally repressed. During strong afferent synaptic stimulation, glutamate (Glu) stimulates both postsynaptic AMPAR and NMDARs. The increase in postsynaptic Ca^2+^ through the activated NMDAR stimulates multiple effector molecules that upregulate PKMζ synthesis and downregulate PKMζ degradation. The newly translated PKMζ is rapidly phosphorylated by PDK1 to achieve a fully active state. PKMζ enhances its own translation by phosphorylating PIN1. The persistent activity of PKMζ then maintains both increases in postsynaptic GluA2-containing AMPARs by decreasing receptor endocytosis through an NSF-dependent pathway, and increases in PSD-95 aggregation. Adapted from [59].

The inhibition of PKMζ persistently disrupts memory storage, rather than transiently blocking memory retrieval [22]. The half-life of intracranially injected ZIP is ~2 hours, and is cleared from the brain within a day [32], but the disruption of previously stored memory by the agent lasts far longer. After bolus injections of ZIP, LTP *in vivo* is eliminated for days [21] and well-established memories are eliminated for at least 1 week in hippocampus [22] and for 1 month in neocortex [24], the longest time points examined in each region. After ZIP has cleared, new memories can nonetheless be reformed and stored [22,27,39], and even erased a second time by ZIP [26]. These data indicate that transiently inhibiting PKMζ does not damage the hippocampus or neocortex, but specifically erases the long-term memory trace maintained by these structures.

Because the half-life of ZIP is ~2 hours [32], initial studies on the disruption of fear conditioning that had tested memory retention a day after injection of the drug into the BLA had indicated that the persistence of memory erasure in the BLA would be similar to that in hippocampus and neocortex [30]. One paper, using a dose of ZIP lower than that employed in previous studies of other brain regions, suggested that the memory loss for fear-potentiated startle was temporary [60], although see the discussions in references [61,62]. A subsequent study of the retention of a learned active avoidance response using the standard dose of ZIP in the BLA, however, confirmed persistent amnesia for a week after drug injection [31], demonstrating that memory erasure by ZIP in the BLA was consistent with that observed in other brain regions. Interestingly, in the study that had used the low dose of ZIP, memory loss persisted when the rats were reexposed to the CS alone, a day after drug infusion [60]. Because the standard dose of ZIP erases multiple memories within a brain region, with or without CS reactivation [24], if low dose ZIP selectively disrupts the CS-US association of the specific reactivated CS, such doses of the drug might be used to erase specific memories, similar to the specific disruption of reactivated memory that is the hallmark of reconsolidation blockade [63].

## The molecular mechanisms of synaptic memory storage by PKMζ

PKMζ’s ability to store experience-dependent information at synapses is due to its unique structure as an autonomously active form of PKC [64-66]. Full-length PKC isoforms are activated by conformational changes induced by second messengers [67]. Each PKC consists of an N-terminal regulatory domain linked by a hinge region to a C-terminal catalytic domain. Under basal conditions in the cell cytosol, full-length PKCs are inactive because an autoinhibitory pseudosubstrate within the regulatory domain interacts with and blocks the catalytic domain. Second messengers stimulate the full-length PKCs by binding to the regulatory domain, translocating the enzyme to membrane, and inducing a conformational change that releases the autoinhibition. This allosteric mechanism activates all three classes of PKC isoforms — *conventional* PKCs by Ca^2+^ and diacylglycerol (DAG); *novel* PKCs by DAG, but not Ca^2+^; and *atypical* PKCs, including the full-length PKCζ, by neither Ca^2+^ nor DAG, but by alternate lipid second messengers and proteins that bind to the aPKC regulatory domain. Because the second messengers that activate the full-length PKCs are generally short-lived, this mechanism of action is transient and rapidly reversible, allowing PKC to participate in multiple rounds of short-term signal transduction.

In contrast to the full-length isoforms, PKMζ consists of a PKC catalytic domain without a regulatory domain [64,65]. Lacking the autoinhibitory pseudosubstrate of the PKCζ regulatory domain (i.e., the amino acid sequence of ZIP), PKMζ is autonomously and thus persistently active [64,66]. The formation of PKM was originally described in biochemical *in vitro* studies to be through limited proteolysis of full-length PKC at its hinge region, separating the regulatory from the catalytic domain [68]. However, the generation of PKMζ in neurons is by a transcriptional and translational mechanism unique to the ζ gene, *PRKCZ*, which produces the ζ catalytic domain directly through new protein synthesis [65] (Figure 1). The *PRKCZ* gene contains two promoters, one upstream of the exons of the N-terminal regulatory domain that generates the full-length PKCζ mRNA and protein, and a second internal promoter within a large intron that produces a PKMζ mRNA, the translation of which begins at an evolutionarily conserved methionine in the hinge region to produce an independent ζ catalytic domain [65]. In the forebrain, PKMζ mRNA is expressed by the *PRKCZ* gene, and PKCζ mRNA is transcribed only in trace amounts, except in the lateral olfactory tract; in the hindbrain, both mRNA species are transcribed [65,69]. Outside the nervous system, PKCζ mRNA is expressed in various cell-types, and PKMζ mRNA is transcribed only in trace amounts [65].

Under basal conditions in neurons, PKMζ mRNA is transported to dendrites [70] and is translationally repressed by its long 5’untranslated region [65]. Following NMDAR activation that triggers LTP, many of the signaling molecules important for LTP induction, including CaMKII, PKA, MAPK, phosphatidylinositol 3-kinase (PI3K), mammalian target of rapamycin (mTOR), as well as actin filament formation, act in concert to increase PKMζ synthesis [65,71,72] (Figure 1). Brain-derived neurotrophic factor (BDNF) injected into the hippocampus *in vivo* also increases PKMζ [73] and in theta burst-triggered LTP plays an additional role in decreasing the initial degradation of PKMζ, thus further contributing to increases in the kinase [74]. Immediately after translation, the nascent PKMζ is rapidly phosphorylated by phosphoinositide-dependent kinase 1 (PDK1), which locks the PKMζ in a maximally activated state [23,71]. Subsequently, persistent PDK1 phosphorylation is no longer required for the autonomous activity of PKMζ [23]. Whereas increased translation of pre-existing PKMζ mRNA is the mechanism for the formation of PKMζ in LTP [65,71], persistent increases in ζ mRNA also occur after some forms of learning [75], suggesting additional transcriptional regulation of the abundance of the PKMζ message and thus the translational capacity of the neuron to synthesize PKMζ. After synthesis, PKMζ acts as an LTP-specific plasticity-related protein (PRP) that is captured at recently activated synapses that have undergone “synaptic tagging” [17,20,76-79]. CaMKII has been proposed to be a component of the synaptic tag sequestering PKMζ [79].

Once at the synapse, the functional target of PKMζ for synaptic potentiation is the GluA2 subunit of the AMPAR [30,58]. The interaction between PKMζ and GluA2, originally described in rodents, is likely to be evolutionarily conserved, because the colocalization of the two molecules at synaptic sites has recently been observed to positively correlate with memory performance in young and aged non-human primates [80]. Interactions between the trafficking protein N-ethylmaleimide-sensitive factor (NSF) and GluA2, which was originally described as part of a homeostatic mechanism maintaining AMPARs at synapses [81-84], are critical for the synaptic potentiation by PKMζ, but the precise targets of phosphorylation that mediate the enhancement have not yet been established [30,58]. PKMζ also interacts with the postsynaptic scaffolding protein, kidney and brain expressed protein (KIBRA) [85,86], which has been associated by genetic studies with human memory performance [87], and the C-terminal of PKMζ is a PSD-95/DLG/ZO-1 (PDZ)-binding sequence that interacts with protein interacting with PKC 1 (PICK1) [58]. Both KIBRA and PICK1 also bind to the AMPAR GluA2 subunit and participate in the regulation of the trafficking of the receptor to postsynaptic sites [88-90].

Perhaps related to its role in AMPAR trafficking, PKMζ also increases the aggregation of postsynaptic density protein 95 (PSD-95) at synapses [53], which may be through phosphorylation of the palmitoylation enzyme ZDHHC8 [91]. PKMζ alters the morphology of spines in cultured neurons [92], and the amount of PKMζ in spines positively correlates with the area of the PSD in synapses of the dentate gyrus in non-human primates [80]. Because ZIP reverses the PKMζ-mediated aggregation of PSD-95 within hours of drug application, these structural changes of synapses may, like synaptic potentiation, be maintained by the persistent enzymatic action of PKMζ [53].

## The evolutionary history of PKMζ, LTP, and long-term memory

A comparative genomic analysis of atypical PKC performed by Wayne Sossin and colleagues at McGill University found that the translational mechanism for the formation of PKMζ, the hallmark of which is a conserved methionine in the hinge region that initiates the synthesis of PKMζ [65], arose around the time of the gene duplication of the single invertebrate aPKC gene into the two vertebrate aPKC isoforms, ζ and ι/λ [93]. These two isoforms, whose actions can be similar in neurons [47], are the two most closely related genes of the 9-member PKC gene family. Extending this analysis, Ling Pan (SUNY Downstate) and I found that the lamprey, an early, jawless cyclostome vertebrate, has an apparent single aPKC, with features of both PKCζ and PKCι/λ, that contains the hallmark hinge methionine found in PKMζ that initiates translation of the independent catalytic domain. Therefore, the formation of atypical PKM by new protein synthesis originated at or before the splitting of cyclostomes from the main vertebrate line of evolution (the cyclostome–gnathostome split). This establishes the origin of the formation of PKM by new protein synthesis, and therefore the mechanism maintaining late-LTP, at least ~500 million years ago in the Cambrian period [94,95].

Remarkably, a persistently active PKM form is also generated from the invertebrate aPKC, which lacks the vertebrate PKMζ translational start site [93], and this atypical PKM plays fundamental roles in long-term memory maintenance in widely divergent invertebrate phyla. Working with the arthropod *Drosophila melanogaster*, Jerry Yin and our colleagues at the University of Wisconsin at Madison showed that the persistent activity of atypical PKM is both necessary and sufficient for long-term memory of olfactory avoidance behavior that is induced by associative conditioning [96]. *Drosophila* atypical PKM is enriched in the fly head [96], just as PKMζ is specifically expressed in neural tissue [65,97], but the mechanism for the formation of atypical PKM in *Drosophila* has not yet been elucidated. In the mollusk *Aplysia californica*, David Glanzman and colleagues at UCLA found that the persistent activity of atypical PKM is crucial for maintaining behavioral long-term sensitization of withdrawal reflexes as late as 7 days after training, well beyond the initial, protein synthesis-dependent consolidation phase for the sensitization [98]. In addition, Glanzman found that the *Aplysia* orthologue of PKMζ also maintains the long-term synaptic facilitation of sensorimotor synapses that mediates the behavior [98]. As shown by Sossin and colleagues, proteolysis of aPKC is critical for the formation of atypical PKM in *Aplysia*, and the proteolytic formation of atypical PKM by sensitizing stimulation requires both the protease calpain and new protein synthesis [93,99]. How long-term memory maintained by atypical PKM in *Aplysia* might require both new protein synthesis and proteolysis is not yet known, but possibilities include new synthesis of the precursor aPKC, of the protease that cleaves the aPKC, or of another molecule that facilitates the cleavage or stabilizes the atypical PKM [99]. Eric Kandel and his colleagues at Columbia University have shown that the translation factor, *Aplysia* cytoplasmic polyadenylation element binding protein (CPEB) that has prion-like properties of self-perpetuation [100,101] is required for sustaining long-term facilitation during a persistent, protein synthesis-dependent period lasting ~ 2 days [102]. Because *Aplysia* atypical PKM also maintains long-term facilitation during this period [98], CPEB may interact with atypical PKM, either by regulating the synthesis of aPKC or the protease that cleaves this precursor to PKM, or, conversely, as a mechanism regulated by PKM.

In both *Drosophila* and rats, overexpression of PKMζ enhances long-term memory. Jerry Yin and our colleagues demonstrated that transgenic flies overexpressing either mouse PKMζ or the *Drosophila* atypical PKM have stronger long-term memory, and therefore the mechanism for memory enhancement by increasing PKMζ activity, like that of memory erasure by decreasing PKMζ activity, is evolutionarily conserved [96]. Furthermore, by transfecting PKMζ into the neocortex of rats, Yadin Dudai and our colleagues at the Weizmann Institute showed that not only are new memories strengthened when PKMζ is overexpressed before training, but even old, faded memories are robustly enhanced when the kinase is overexpressed a week after training [25]. The mechanisms by which increasing PKMζ by overexpression enhances memory in both vertebrates and invertebrates are not known, but may involve upregulation of the positive feedback loops of local translation and “synaptic autotagging” that have been proposed to maintain the synaptic compartmentalization of PKMζ [59], as discussed in the next section.

**Figure 2:**
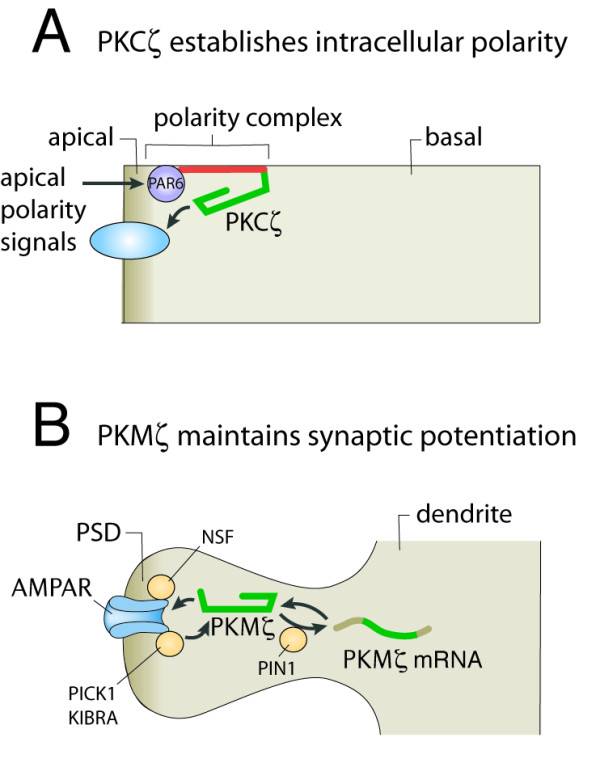
**Model of PKMζ-mediated LTP maintenance as a specialized form of aPKC regulation of cell polarity.A**) In polarized cells such as epithelial cells, polarity signals activate PAR6, which binds to the aPKC regulatory domain (red) and activates the enzyme. Phosphorylation by aPKC then traffics membrane proteins to the apical compartment of the polarized cell. **B**) In spines, PKMζ is synthesized after LTP induction or learning and potentiates synaptic strength by NSF-dependent trafficking of AMPARs to the PSD, the apical compartment of the postsynaptic spine. The absence of a PKCζ regulatory domain isolates PKMζ from other postsynaptic signaling, allowing the kinase to store long-term information without interference from short-term synaptic events. PKMζ maintains both synaptic potentiation and its own localization at the synapse by forming positive feedback loops, involving binding of PKMζ to postsynaptic GluA2 subunit-containing AMPAR-binding proteins, such as PICK1 and KIBRA. The persistent activity of postsynaptic PKMζ is required to maintain decreased AMPAR endocytosis, preventing both AMPAR and kinase elimination from the potentiated synapse. Other positive feedback loops, such as that involving PIN1, maintain increases in the amount of PKMζ through enhanced local translation.

Why is the persistently active PKM form of an atypical PKC crucial for memory maintenance, whether it is generated by cleavage of full-length PKC as in *Aplysia*, or by transcription from an internal promoter within the PKCζ gene as in vertebrates? Although one can only speculate, a clue may be the original function of aPKC in cells. Single cell organisms such as yeast express a single PKC, but multicellular animals express multiple PKC isoforms generated by gene duplication. In *C. elegans*, the function of aPKC has already specialized to establish and maintain apical compartments within polarized cells through participation in a highly conserved multiprotein complex, called the anterior PAR complex (for *par*titioning), consisting of the adapter proteins PAR6 and PAR3, the small GTPase Cdc42, and aPKC [103] (Figure 2A). In this apically localized complex, Cdc42 receives extracellular signals and stimulates PAR6, which then binds to the regulatory domain of aPKC, activating the kinase [104]. The PAR complex is conserved in polarized cells throughout evolution and defines the anterior pole of the *C. elegans* embryo, the apical domain of *Drosophila* neuroblasts to control their asymmetric division, and the apical membrane of epithelial cells to promote apical-basal polarity and the formation and maintenance of cell–cell junctions [103,105-107]. Although the mechanisms by which the PAR complex mediate polarity are only beginning to be elucidated, a genome-wide screen in *C. elegans* has shown that the complex directs the trafficking of membrane proteins through the regulation of endocytosis and vesicle recycling [108,109]. This mechanism is evolutionarily conserved because it is also observed in human HeLa cells [108].

The general function of aPKC to distribute membrane proteins to apical compartments may have adapted to control the trafficking of glutamate receptors to the postsynaptic density, the apical compartment of the synaptic spine (Figure 2B). Atypical PKC may originally have participated in development of the synapse. Indeed, roles for PKMζ in synaptic maturation and dendritic development have recently been described [91,110].

Once established as a mechanism for trafficking glutamate receptors to the synapse during development, the further activation of full-length aPKC might have been useful for short-term synaptic plasticity and short-term memory. Then, mutations that either allow proteolysis in the hinge between the regulatory and catalytic domains in invertebrates [99], or that generate independent translation of the catalytic domain in vertebrates [65], would have transformed this short-term memory mechanism into a long-term memory mechanism (Figure 2B).

The truncation of the aPKC regulatory domain to form an independent catalytic domain would serve two purposes in a molecular mechanism of long-term memory (Figure 2B). First, the enzymatic activity of aPKC becomes persistent, because of the removal of the autoinhibitory pseudosubstrate of the regulatory domain, as described above. Second, the regulation of this persistent atypical PKM activity becomes functionally isolated from the extracellular signaling that is normally transmitted into the cell by the other PAR proteins and second messengers that activate the full-length kinase by binding to the aPKC regulatory domain. Thus, once formed, the autonomous activity of atypical PKM that maintains long-term memory is independent from the transient signal transduction events that regulate short-term synaptic potentiation or depression. This feature may be important if long-term information about experiences in the past is to be stored in the same neural circuitry that is continually modified by short-term experiences in the present.

## Making and breaking PKMζ-mediated positive feedback loops to maintain and erase long-term memory

Because memories up to 3 months old can be erased by PKMζ inhibitors [26] and the amnestic effect of PKMζ inhibition is blocked by GluA2 endocytosis inhibitors [30,34,36], a recent review has proposed that PKMζ and its downstream targets form a self-perpetuating, positive-feedback network through a process of “synaptic auto-tagging” that can persist for months to maintain very long-term memories [59] (Figure 2B). After its local synthesis during LTP or memory formation, PKMζ, by a process involving inhibition of endocytosis through the action of NSF, traffics GluA2-containing AMPARs to the synapse, where AMPAR-binding proteins, such as PICK1 and KIBRA, also bind to PKMζ and thus maintain the kinase at the appropriate postsynaptic sites. Applications of PKMζ inhibitors disrupt the positive-feedback signaling network and erase the potentiated state of the synapse and long-term memory by releasing the GluA2 endocytosis that has been inhibited by the activity of PKMζ. Thus, PKMζ inhibition allows the AMPARs and PKMζ to be eliminated from the appropriate postsynaptic sites. The collapse of the positive feedback network would be rapid, because both LTP [2] and long-term memory [22,27] are disrupted within 2 hours of exposure to ZIP or other PKMζ inhibitors. After the inhibitors have been eliminated, because the original postsynaptic sites of PKMζ formation and subsequent positive feedback have been lost, the potentiation [21] and long-term memory [22,24] are permanently eliminated, and the strength of the synapse and the animal’s behavior reset to the naïve state [59].

Other positive feedback loops involving enhanced local translation have been proposed to maintain increased levels of the kinase at potentiated synapses (Figure 2B). In LTP, for example, PKMζ phosphorylation is required for the increased synthesis of PKMζ [71]. In particular, PKMζ phosphorylation of peptidyl-prolyl cis-trans isomerase NIMA-interacting 1 (PIN1) upregulates local dendritic synthesis and the translation of PKMζ [111] (Figures 1 and 2B). Interestingly, phosphorylation by the *Aplysia* atypical PKM is required for the formation of the kinase by proteolysis, also forming a positive feedback loop [99]. Thus, different positive feedback loops may have evolved to maintain persistent increased atypical PKM in vertebrates and invertebrates, each specific to the mechanism by which the atypical PKM is generated during the formation of long-term memory.

Although the long-term storage of information by PKMζ is isolated from short-term signaling at the synapse, as discussed in the previous section, the rapid erasure of memory by PKMζ inhibitors suggests the possibility that the long-term information stored by PKMζ might also be modifiable by new experiences. Thus, an animal might quickly update a long-term memory by rapidly degrading the PKMζ molecules restricted to potentiated synapses and disrupting the positive feedback loops maintaining the maladaptive information. The physiological erasure of a long-term memory within a neural circuit by downregulating PKMζ would return the circuit to its naïve state, while preserving the circuitry that had been established during development to mediate specific behaviors. This is because whereas pharmacological or dominant negative inhibitors of PKMζ disrupt LTP maintenance, these inhibitors do not affect basal synaptic transmission either in brain slices or *in vivo* in mature animals [16,19,22,23,57].

The downregulation of persistent increases of PKMζ has recently been observed during memory extinction and reconsolidation blockade [112]. In animal models of drug addiction, persistent increases of PKMζ maintain drug-craving memory in the neural circuitry mediating reward and emotion, including the nucleus accumbens and the BLA [34,38,112]. When memories associated with drug use are partially extinguished by repetitive exposure to the CS, the previously induced, persistent increases of PKMζ in the BLA are reversed, and PKMζ increases in the infralimbic cortex, where the kinase is critical for maintaining extinction [38]. Furthermore, a form of reconsolidation blockade, in which reactivation of the memory is followed by extinction, produces more robust reversal of PKMζ in the BLA, greater increases in the infralimbic cortex, and stronger disruption of the drug-associated memory than extinction alone [112]. Decreases in PKMζ have also been observed in the hippocampus with spatial familiarity [113], which, like extinction, is produced by multiple exposures to an environment without experimental reinforcement. Although the mechanism for downregulating PKMζ during the repetitive exposure to a stimulus is not known, proteolytic degradation of PKMζ has been observed in the maintenance of NMDAR-dependent long-term depression (LTD) [114,115], suggesting the possibility that LTD or depotentiation induces a persistent loss of PKMζ that maintains familiarity and, in some circuits, the extinction of memory.

Fundamental information for understanding these mechanisms of memory stability and erasure are the half-life of PKMζ and the mechanisms maintaining the compartmentalization of the kinase at specific synapses, such as those proposed in the model of PKMζ synaptic auto-tagging [59]. Although another mechanism of memory storage may take over from PKMζ after 3 months [26], the more parsimonious hypothesis is that PKMζ maintains information for a memory’s lifetime. For humans, how can the fidelity of PKMζ-mediated positive feedback loops be maintained for decades? What is the relationship between the persistence of PKMζ that functionally maintains long-term memory and the structural changes associated with long-term memory, particularly the growth of new synapses [7]? These are essential questions for the future study of PKMζ and memory storage.

## Conclusion

The persistent increased activity of PKMζ maintains LTP and perpetuates many, but not all, forms of long-term memory. PKMζ and its invertebrate orthologues provide insight into the evolutionary history of LTP-like synaptic plasticity and long-term memory. The role of atypical PKM in maintaining long-term memory may have emerged as a specialized mechanism for persistently increasing postsynaptic AMPARs from the more general function of aPKC in membrane protein trafficking to the apical compartment of polarized cells. The origin of the mechanism of late-LTP by new synthesis of atypical PKM can be traced to early vertebrates in the Cambrian period. This synaptic information storage mechanism proved capable of recording experiences within neural circuits in a way that was both stable for months, yet reversible as new contingencies arise, and appears to have been useful for animal survival for hundreds of millions of years.

## Competing interests

The author has no competing financial interests.

## References

[B1] Cajal R La fine structure des centres nerveux Proc R Soc Lond 1894 55 444 468 10.1098/rspl.1894.0063

[B2] Kandel ER Schwartz JH Molecular biology of learning: modulation of transmitter release Science 1982 218 4571 433 443 10.1126/science.6289442 6289442

[B3] Dudai Y Neurogenetic dissection of learning and short-term memory in Drosophila Annu Rev Neurosci 1988 11 537 563 10.1146/annurev.ne.11.030188.002541 3129981

[B4] Dash PK Hochner B Kandel ER Injection of the cAMP-responsive element into the nucleus of Aplysia sensory neurons blocks long-term facilitation Nature 1990 345 6277 718 721 10.1038/345718a0 2141668

[B5] Yin JC Wallach JS Del Vecchio M Wilder EL Zhou H Quinn WG Tully T Induction of a dominant negative CREB transgene specifically blocks long-term memory in Drosophila Cell 1994 79 1 49 58 10.1016/0092-8674(94)90399-9 7923376

[B6] Silva AJ Kogan JH Frankland PW Kida S CREB and memory Annu Rev Neurosci 1998 21 127 148 10.1146/annurev.neuro.21.1.127 9530494

[B7] Kandel ER The molecular biology of memory storage: a dialogue between genes and synapses Science 2001 294 5544 1030 1038 10.1126/science.1067020 11691980

[B8] Lømo T Frequency potentiation of excitatory synaptic activity in the dentate area of the hippocampal formation Acta Physiol Scand 1966 68 Suppl 128

[B9] Bliss TVP Lømo T Long-lasting potentiation of synaptic transmission in the dentate area of the anaesthetized rabbit following stimulation of the perforant path J Physiol 1973 232 331 356 4727084 10.1113/jphysiol.1973.sp010273 PMC1350458

[B10] Collingridge GL Kehl SJ McLennan H Excitatory amino acids in synaptic transmission in the Schaffer collateral-commissural pathway of the rat hippocampus J Physiol 1983 334 34 46 10.1113/jphysiol.1983.sp014478 PMC1197298 6306230

[B11] Morris RG Anderson E Lynch GS Baudry M Selective impairment of learning and blockade of long-term potentiation by an N-methyl-D-aspartate receptor antagonist, AP5 Nature 1986 319 6056 774 776 10.1038/319774a0 2869411

[B12] Sanes JR Lichtman JW Can molecules explain long-term potentiation? Nat Neurosci 1999 2 7 597 604 10.1038/10154 10404178

[B13] Lisman J Malenka RC Nicoll RA Malinow R Learning mechanisms: the case for CaM-KII Science 1997 276 5321 2001 2002 10.1126/science.276.5321.2001 9221509

[B14] English JD Sweatt JD A requirement for the mitogen-activated protein kinase cascade in hippocampal long term potentiation J Biol Chem 1997 272 31 19103 19106 10.1074/jbc.272.31.19103 9235897

[B15] Kelleher RJ Govindarajan A Jung HY Kang H Tonegawa S Translational control by MAPK signaling in long-term synaptic plasticity and memory Cell 2004 116 467 479 10.1016/S0092-8674(04)00115-1 15016380

[B16] Ling DS Benardo LS Serrano PA Blace N Kelly MT Crary JF Sacktor TC Protein kinase Mζ is necessary and sufficient for LTP maintenance Nat Neurosci 2002 5 4 295 296 10.1038/nn829 11914719

[B17] Frey U Morris RG Synaptic tagging and long-term potentiation Nature 1997 385 6616 533 536 10.1038/385533a0 9020359

[B18] Davis HP Squire LR Protein synthesis and memory: a review Psychol Bull 1984 96 518 559 6096908

[B19] Serrano P Yao Y Sacktor TC Persistent phosphorylation by protein kinase Mζ maintains late-phase long-term potentiation J Neurosci 2005 25 8 1979 1984 10.1523/JNEUROSCI.5132-04.2005 15728837 PMC6726070

[B20] Sajikumar S Navakkode S Sacktor TC Frey JU Synaptic tagging and cross-tagging: the role of protein kinase Mζ in maintaining long-term potentiation but not long-term depression J Neurosci 2005 25 24 5750 5756 10.1523/JNEUROSCI.1104-05.2005 15958741 PMC6724879

[B21] Madronal N Gruart A Sacktor TC Delgado-Garcia JM PKMζ inhibition reverses learning-induced increases in hippocampal synaptic strength and memory during trace eyeblink conditioning PLoS One 2010 5 4 e10400 10.1371/journal.pone.0010400 20454458 PMC2861600

[B22] Pastalkova E Serrano P Pinkhasova D Wallace E Fenton AA Sacktor TC Storage of spatial information by the maintenance mechanism of LTP Science 2006 313 5790 1141 1144 10.1126/science.1128657 16931766

[B23] Yao Y Shao C Jothianandana D Tcherepanova A Shouval H Sacktor T Matching biochemical and functional efficacies confirm ZIP as a potent competitive inhibitor of PKMζ in neurons Neuropharmacology 2012 http://dx.doi.org/10.1016/j.neuropharm.2012.07.018 10.1016/j.neuropharm.2012.07.018 PMC3445653 22846225

[B24] Shema R Sacktor TC Dudai Y Rapid erasure of long-term memory associations in cortex by an inhibitor of PKMζ Science 2007 317 5840 951 953 10.1126/science.1144334 17702943

[B25] Shema R Haramati S Ron S Hazvi S Chen A Sacktor TC Dudai Y Enhancement of consolidated long-term memory by overexpression of protein kinase Mzeta in the neocortex Science 2011 331 6021 1207 1210 10.1126/science.1200215 21385716

[B26] Shema R Hazvi S Sacktor TC Dudai Y Boundary conditions for the maintenance of memory by PKMζ in neocortex Learn Mem 2009 16 2 122 128 10.1101/lm.1183309 19181618 PMC2661244

[B27] Serrano P Friedman EL Kenney J Taubenfeld SM Zimmerman JM Hanna J Alberini C Kelley AE Maren S Rudy JW PKMζ maintains spatial, instrumental, and classically conditioned long-term memories PLoS Biol 2008 6 12 2698 2706 19108606 10.1371/journal.pbio.0060318 PMC2605920

[B28] Hardt O Migues PV Hastings M Wong J Nader K PKMzeta maintains 1-day- and 6-day-old long-term object location but not object identity memory in dorsal hippocampus Hippocampus 2010 20 6 691 695 19806657 10.1002/hipo.20708

[B29] Kwapis JL Jarome TJ Lonergan ME Helmstetter FJ Protein kinase Mzeta maintains fear memory in the amygdala but not in the hippocampus Behav Neurosci 2009 123 4 844 850 19634944 10.1037/a0016343 PMC2782955

[B30] Migues PV Hardt O Wu DC Gamache K Sacktor TC Wang YT Nader K PKMζ maintains memories by regulating GluR2-dependent AMPA receptor trafficking Nat Neurosci 2010 13 5 630 634 10.1038/nn.2531 20383136

[B31] Gamiz F Gallo M Intra-amygdala ZIP injections impair the memory of learned active avoidance responses and attenuate conditioned taste-aversion acquisition in rats Learn Mem 2011 18 8 529 533 10.1101/lm.2253311 21784922

[B32] Kwapis JL Jarome TJ Gilmartin MR Helmstetter FJ Intra-amygdala infusion of the protein kinase Mzeta inhibitor ZIP disrupts foreground context fear memory Neurobiol Learn Mem 2012 98 2 148 153 10.1016/j.nlm.2012.05.003 22659643 PMC3424353

[B33] Crespo JA Stockl P Ueberall F Jenny M Saria A Zernig G Activation of PKCzeta and PKMzeta in the nucleus accumbens core is necessary for the retrieval, consolidation and reconsolidation of drug memory PLoS One 2012 7 2 e30502 10.1371/journal.pone.0030502 22348011 PMC3277594

[B34] Li YQ Xue YX He YY Li FQ Xue LF Xu CM Sacktor TC Shaham Y Lu L Inhibition of PKMzeta in nucleus accumbens core abolishes long-term drug reward memory J Neurosci 2011 31 14 5436 5446 10.1523/JNEUROSCI.5884-10.2011 21471379 PMC3150199

[B35] Shabashov D Shohami E Yaka R Inactivation of PKMzeta in the NAc Shell Abolished Cocaine-Conditioned Reward J Mol Neurosci 2012 47 3 546 553 10.1007/s12031-011-9671-7 22127928

[B36] Pauli WM Clark AD Guenther HJ O'Reilly RC Rudy JW Inhibiting PKMzeta reveals dorsal lateral and dorsal medial striatum store the different memories needed to support adaptive behavior Learn Mem 2012 19 7 307 314 10.1101/lm.025148.111 22723053

[B37] Sacco T Sacchetti B Role of secondary sensory cortices in emotional memory storage and retrieval in rats Science 2010 329 5992 649 656 10.1126/science.1183165 20689011

[B38] He YY Xue YX Wang JS Fang Q Liu JF Xue LF Lu L PKMzeta maintains drug reward and aversion memory in the basolateral amygdala and extinction memory in the infralimbic cortex Neuropsychopharmacology 2011 36 10 1972 1981 10.1038/npp.2011.63 21633338 PMC3158313

[B39] von Kraus LM Sacktor TC Francis JT Erasing sensorimotor memories via PKMzeta inhibition PLoS One 2010 5 6 e11125 10.1371/journal.pone.0011125 20559553 PMC2886075

[B40] Cooke SF Bear MF Visual experience induces long-term potentiation in the primary visual cortex J Neurosci 2010 30 48 16304 16313 10.1523/JNEUROSCI.4333-10.2010 21123576 PMC3078625

[B41] Barry J Rivard B Fox S Fenton A Sacktor T Muller R Inhibition of PKMζ disrupts the stable spatial discharge of hippocampal place cells in a familiar environment J Neurosciin press 10.1523/JNEUROSCI.0319-12.2012 PMC3752127 23035087

[B42] Li XY Ko HG Chen T Descalzi G Koga K Wang H Kim SS Shang Y Kwak C Park SW Alleviating neuropathic pain hypersensitivity by inhibiting PKMzeta in the anterior cingulate cortex Science 2010 330 6009 1400 1404 10.1126/science.1191792 21127255

[B43] King T Qu C Okun A Melemedjian OK Mandell EK Maskaykina IY Navratilova E Dussor GO Ghosh S Price TJ Contribution of PKMzeta-dependent and independent amplification to components of experimental neuropathic pain Pain 2012 153 6 1263 1273 10.1016/j.pain.2012.03.006 22482911 PMC3358498

[B44] Li XY Ko HG Chen T Collingridge GL Kaang BK Zhuo M Erasing injury-related cortical synaptic potentiation as a new treatment for chronic pain J Mol Med (Berl) 2011 89 9 847 855 10.1007/s00109-011-0768-9 21584648

[B45] Asiedu MN Tillu DV Melemedjian OK Shy A Sanoja R Bodell B Ghosh S Porreca F Price TJ Spinal protein kinase M zeta underlies the maintenance mechanism of persistent nociceptive sensitization J Neurosci 2011 31 18 6646 6653 10.1523/JNEUROSCI.6286-10.2011 21543593 PMC3090264

[B46] Laferriere A Pitcher MH Haldane A Huang Y Cornea V Kumar N Sacktor TC Cervero F Coderre TJ PKMzeta is essential for spinal plasticity underlying the maintenance of persistent pain Mol Pain 2011 7 99 10.1186/1744-8069-7-99 22185613 PMC3266216

[B47] Zhang YH Kays J Hodgdon KE Sacktor TC Nicol GD Nerve growth factor enhances the excitability of rat sensory neurons through activation of the atypical protein kinase C isoform, PKMzeta J Neurophysiol 2012 107 1 315 335 10.1152/jn.00030.2011 21975456 PMC3349696

[B48] Marchand F D'Mello R Yip PK Calvo M Muller E Pezet S Dickenson AH McMahon SB Specific involvement of atypical PKCzeta/PKMzeta in spinal persistent nociceptive processing following peripheral inflammation in rat Mol Pain 2011 7 86 10.1186/1744-8069-7-86 22054645 PMC3253059

[B49] Cohen H Kozlovsky N Matar MA Kaplan Z Zohar J Mapping the brain pathways of traumatic memory: inactivation of protein kinase M zeta in different brain regions disrupts traumatic memory processes and attenuates traumatic stress responses in rats Eur Neuropsychopharmacol 2010 20 4 253 271 10.1016/j.euroneuro.2009.12.006 20129764

[B50] Monti MC Gabach LA Perez MF Ramirez OA Impact of contextual cues in the expression of the memory associated with diazepam withdrawal: involvement of hippocampal PKMzeta in vivo, and Arc expression and LTP in vitro Eur J Neurosci 2012 10.1111/j.1460-9568.2012.08206.x 22759216

[B51] Ho SY Chen CH Liu TH Chang HF Liou JC Protein kinase mzeta is necessary for cocaine-induced synaptic potentiation in the ventral tegmental area Biol Psychiatry 2012 71 8 706 713 10.1016/j.biopsych.2011.10.031 22153887

[B52] Crary JF Shao CY Mirra SS Hernandez AI Sacktor TC Atypical protein kinase C in neurodegenerative disease I: PKMζ aggregates with limbic neurofibrillary tangles and AMPA receptors in Alzheimer disease J Neuropathol Exp Neurol 2006 65 4 319 326 10.1097/01.jnen.0000218442.07664.04 16691113

[B53] Shao CY Sondhi R van de Nes PS Sacktor TC PKMζ is necessary and sufficient for synaptic clustering of PSD-95 Hippocampus 2011 22 7 1501 1507 22378468 10.1002/hipo.20996 PMC3371310

[B54] Laudanna C Mochly-Rosen D Liron T Constantin G Butcher EC Evidence of zeta protein kinase C involvement in polymorphonuclear neutrophil integrin-dependent adhesion and chemotaxis J Biol Chem 1998 273 46 30306 30315 10.1074/jbc.273.46.30306 9804792

[B55] Suzuki A Hirata M Kamimura K Maniwa R Yamanaka T Mizuno K Kishikawa M Hirose H Amano Y Izumi N aPKC acts upstream of PAR-1b in both the establishment and maintenance of mammalian epithelial polarity Curr Biol 2004 14 16 1425 1435 10.1016/j.cub.2004.08.021 15324659

[B56] Wu-Zhang AX Schramm CL Nabavi S Malinow R Newton AC Cellular Pharmacology of Protein Kinase Mzeta (PKMzeta) Contrasts with Its in Vitro Profile: Implications for PKMzeta as a mediator of memory J Biol Chem 2012 287 16 12879 12885 10.1074/jbc.M112.357244 22378786 PMC3339930

[B57] Ling DS Benardo LS Sacktor TC Protein kinase Mζ enhances excitatory synaptic transmission by increasing the number of active postsynaptic AMPA receptors Hippocampus 2006 16 5 443 452 10.1002/hipo.20171 16463388

[B58] Yao Y Kelly MT Sajikumar S Serrano P Tian D Bergold PJ Frey JU Sacktor TC PKMζ maintains late long-term potentiation by N-ethylmaleimide-sensitive factor/GluR2-dependent trafficking of postsynaptic AMPA receptors J Neurosci 2008 28 31 7820 7827 10.1523/JNEUROSCI.0223-08.2008 18667614 PMC2597488

[B59] Sacktor TC How does PKMζ maintain long-term memory? Nat Rev Neurosci 2011 12 1 9 15 21119699 10.1038/nrn2949

[B60] Parsons RG Davis M Temporary disruption of fear-potentiated startle following PKMzeta inhibition in the amygdala Nat Neurosci 2011 14 3 295 296 10.1038/nn.2745 21258326 PMC3080103

[B61] Nader K On the Temporary Nature of Disruption of Fear-Potentiated Startle Following PKMzeta Inhibition in the Amygdale Front Behav Neurosci 2011 5 29 21734872 10.3389/fnbeh.2011.00029 PMC3121052

[B62] Parsons RG Davis M Gone but not Forgotten Front Behav Neurosci 2011 5 51 21887139 10.3389/fnbeh.2011.00051 PMC3157672

[B63] Nader K Schafe GE LeDoux JE The labile nature of consolidation theory Nat Rev Neurosci 2000 1 3 216 219 10.1038/35044580 11257912

[B64] Sacktor TC Osten P Valsamis H Jiang X Naik MU Sublette E Persistent activation of the ζ isoform of protein kinase C in the maintenance of long-term potentiation Proc Natl Acad Sci USA 1993 90 18 8342 8346 10.1073/pnas.90.18.8342 8378304 PMC47352

[B65] Hernandez AI Blace N Crary JF Serrano PA Leitges M Libien JM Weinstein G Tcherapanov A Sacktor TC Protein kinase Mζ synthesis from a brain mRNA encoding an independent protein kinase Cζ catalytic domain. Implications for the molecular mechanism of memory J Biol Chem 2003 278 41 40305 40316 10.1074/jbc.M307065200 12857744

[B66] Schwartz JH Cognitive kinases Proc Natl Acad Sci USA 1993 90 18 8310 8313 10.1073/pnas.90.18.8310 8104334 PMC47345

[B67] Nishizuka Y The molecular heterogeneity of protein kinase C and its implication for cellular recognition Nature 1988 334 6184 661 665 10.1038/334661a0 3045562

[B68] Takai Y Kishimoto A Inoue M Nishizuka Y Studies on a cyclic nucleotide-independent protein kinase and its proenzyme in mammalian tissues. I. Purification and characterization of an active enzyme from bovine cerebellum J Biol Chem 1977 252 21 7603 7609 199593

[B69] Oster H Eichele G Leitges M Differential expression of atypical PKCs in the adult mouse brain Brain Res Mol Brain Res 2004 127 1–2 79 88 15306123 10.1016/j.molbrainres.2004.05.009

[B70] Muslimov IA Nimmrich V Hernandez AI Tcherepanov A Sacktor TC Tiedge H Dendritic transport and localization of protein kinase Mζ mRNA: Implications for molecular memory consolidation J Biol Chem 2004 279 50 52613 52622 10.1074/jbc.M409240200 15371429 PMC1828843

[B71] Kelly MT Crary JF Sacktor TC Regulation of protein kinase Mζ synthesis by multiple kinases in long-term potentiation J Neurosci 2007 27 13 3439 3444 10.1523/JNEUROSCI.5612-06.2007 17392460 PMC6672124

[B72] Kelly MT Yao Y Sondhi R Sacktor TC Actin polymerization regulates the synthesis of PKMζ in LTP Neuropharmacology 2006 52 1 41 45 16914172 10.1016/j.neuropharm.2006.07.002

[B73] Adasme T Haeger P Paula-Lima AC Espinoza I Casas-Alarcon MM Carrasco MA Hidalgo C Involvement of ryanodine receptors in neurotrophin-induced hippocampal synaptic plasticity and spatial memory formation Proc Natl Acad Sci USA 2011 108 7 3029 3034 10.1073/pnas.1013580108 21282625 PMC3041089

[B74] Mei F Nagappan G Ke Y Sacktor TC Lu B BDNF facilitates L-LTP maintenance in the absence of protein synthesis through PKMzeta PLoS One 2011 6 6 e21568 10.1371/journal.pone.0021568 21747912 PMC3126837

[B75] Klur S Muller C Pereira de Vasconcelos A Ballard T Lopez J Galani R Certa U Cassel JC Hippocampal-dependent spatial memory functions might be lateralized in rats: An approach combining gene expression profiling and reversible inactivation Hippocampus 2009 19 9 800 816 10.1002/hipo.20562 19235229

[B76] Parvez S Ramachandran B Frey JU Functional differences between and across different regions of the apical branch of hippocampal CA1 dendrites with respect to long-term depression induction and synaptic cross-tagging J Neurosci 2010 30 14 5118 5123 10.1523/JNEUROSCI.5808-09.2010 20371832 PMC6632803

[B77] Sajikumar S Korte M Different compartments of apical CA1 dendrites have different plasticity thresholds for expressing synaptic tagging and capture Learn Mem 2011 18 5 327 331 10.1101/lm.2095811 21511882

[B78] Smolen P Baxter DA Byrne JH Molecular constraints on synaptic tagging and maintenance of long-term potentiation: a predictive model PLoS Comput Biol 2012 8 8 e1002620 10.1371/journal.pcbi.1002620 22876169 PMC3410876

[B79] Sajikumar S Li Q Abraham WC Xiao ZC Priming of short-term potentiation and synaptic tagging/capture mechanisms by ryanodine receptor activation in rat hippocampal CA1 Learn Mem 2009 16 3 178 186 10.1101/lm.1255909 19223601

[B80] Hara Y Punsoni M Yuk F Park CS Janssen WG Rapp PR Morrison JH Synaptic distributions of GluA2 and PKMzeta in the monkey dentate gyrus and their relationships with aging and memory J Neurosci 2012 32 21 7336 7344 10.1523/JNEUROSCI.0605-12.2012 22623679 PMC3391702

[B81] Nishimune A Isaac JT Molnar E Noel J Nash SR Tagaya M Collingridge GL Nakanishi S Henley JM NSF binding to GluR2 regulates synaptic transmission Neuron 1998 21 1 87 97 10.1016/S0896-6273(00)80517-6 9697854

[B82] Osten P Srivastava S Inman GJ Vilim FS Khatri L Lee LM States BA Einheber S Milner TA Hanson PI The AMPA receptor GluR2 C terminus can mediate a reversible, ATP-dependent interaction with NSF and alpha- and beta-SNAPs Neuron 1998 21 1 99 110 10.1016/S0896-6273(00)80518-8 9697855

[B83] Song I Kamboj S Xia J Dong H Liao D Huganir RL Interaction of the N-ethylmaleimide-sensitive factor with AMPA receptors Neuron 1998 21 2 393 400 10.1016/S0896-6273(00)80548-6 9728920

[B84] Luthi A Chittajallu R Duprat F Palmer MJ Benke TA Kidd FL Henley JM Isaac JT Collingridge GL Hippocampal LTD expression involves a pool of AMPARs regulated by the NSF-GluR2 interaction Neuron 1999 24 2 389 399 10.1016/S0896-6273(00)80852-1 10571232

[B85] Yoshihama Y Hirai T Ohtsuka T Chida K KIBRA co-localizes with protein kinase Mzeta (PKMzeta) in the mouse hippocampus Biosci Biotechnol Biochem 2009 73 1 147 151 10.1271/bbb.80564 19129633

[B86] Buther K Plaas C Barnekow A Kremerskothen J KIBRA is a novel substrate for protein kinase Czeta Biochem Biophys Res Commun 2004 317 3 703 707 10.1016/j.bbrc.2004.03.107 15081397

[B87] Papassotiropoulos A Stephan DA Huentelman MJ Hoerndli FJ Craig DW Pearson JV Huynh KD Brunner F Corneveaux J Osborne D Common Kibra alleles are associated with human memory performance Science 2006 314 5798 475 478 10.1126/science.1129837 17053149

[B88] Makuch L Volk L Anggono V Johnson RC Yu Y Duning K Kremerskothen J Xia J Takamiya K Huganir RL Regulation of AMPA receptor function by the human memory-associated gene KIBRA Neuron 2011 71 6 1022 1029 10.1016/j.neuron.2011.08.017 21943600 PMC3200575

[B89] Xia J Zhang X Staudinger J Huganir RL Clustering of AMPA receptors by the synaptic PDZ domain-containing protein PICK1 Neuron 1999 22 1 179 187 10.1016/S0896-6273(00)80689-3 10027300

[B90] Dev KK Nishimune A Henley JM Nakanishi S The protein kinase C alpha binding protein PICK1 interacts with short but not long form alternative splice variants of AMPA receptor subunits Neuropharmacology 1999 38 5 635 644 10.1016/S0028-3908(98)00230-5 10340301

[B91] Yoshii A Murata Y Kim J Zhang C Shokat KM Constantine-Paton M TrkB and protein kinase Mzeta regulate synaptic localization of PSD-95 in developing cortex J Neurosci 2011 31 33 11894 11904 10.1523/JNEUROSCI.2190-11.2011 21849550 PMC3158490

[B92] Ron S Dudai Y Segal M Overexpression of PKMzeta Alters Morphology and Function of Dendritic Spines in Cultured Cortical Neurons Cereb Cortex 2011 10.1093/cercor/bhr323 PMC4705334 22123937

[B93] Bougie JK Lim T Farah CA Manjunath V Nagakura I Ferraro GB Sossin WS The atypical protein kinase C in Aplysia can form a protein kinase M by cleavage J Neurochem 2009 109 4 1129 1143 10.1111/j.1471-4159.2009.06045.x 19302474 PMC5154740

[B94] Donoghue PCJ Forey PL Aldridge RJ Conodont affinity and chordate phylogeny Biol Rev 2000 75 191 251 10881388 10.1017/s0006323199005472

[B95] Donoghue PCJ Smith MP Sansom IJ Donoghue PCJ, Smith MP The origin and early evolution of chordates: molecular clocks and the fossil record Telling the evolutionary time: molecular clocks and the fossil record 2003 CRC Press, London 190 223

[B96] Drier EA Tello MK Cowan M Wu P Blace N Sacktor TC Yin JC Memory enhancement and formation by atypical PKM activity in Drosophila melanogaster Nat Neurosci 2002 5 4 316 324 10.1038/nn820 11914720

[B97] Naik MU Benedikz E Hernandez I Libien J Hrabe J Valsamis M Dow-Edwards D Osman M Sacktor TC Distribution of protein kinase Mζ and the complete protein kinase C isoform family in rat brain J Comp Neurol 2000 426 2 243 258 10.1002/1096-9861(20001016)426:2<243::AID-CNE6>3.0.CO;2-8 10982466

[B98] Cai D Pearce K Chen S Glanzman DL Protein kinase M maintains long-term sensitization and long-term facilitation in Aplysia J Neurosci 2011 31 17 6421 6431 10.1523/JNEUROSCI.4744-10.2011 21525283 PMC3102530

[B99] Bougie JK Cai D Hastings M Farah CA Chen S Fan X McCamphill PK Glanzman DL Sossin WS Serotonin-induced cleavage of the atypical Protein Kinase C Apl III in Aplysia J Neurosciin press 10.1523/JNEUROSCI.3026-11.2012 PMC6621423 23077049

[B100] Si K Giustetto M Etkin A Hsu R Janisiewicz AM Miniaci MC Kim JH Zhu H Kandel ER A neuronal isoform of CPEB regulates local protein synthesis and stabilizes synapse-specific long-term facilitation in Aplysia Cell 2003 115 7 893 904 10.1016/S0092-8674(03)01021-3 14697206

[B101] Si K Lindquist S Kandel ER A neuronal isoform of the Aplysia CPEB has prion-like properties Cell 2003 115 7 879 891 10.1016/S0092-8674(03)01020-1 14697205

[B102] Miniaci MC Kim JH Puthanveettil SV Si K Zhu H Kandel ER Bailey CH Sustained CPEB-dependent local protein synthesis is required to stabilize synaptic growth for persistence of long-term facilitation in Aplysia Neuron 2008 59 6 1024 1036 10.1016/j.neuron.2008.07.036 18817739 PMC3442368

[B103] Suzuki A Ohno S The PAR-aPKC system: lessons in polarity J Cell Sci 2006 119 Pt 6 979 987 16525119 10.1242/jcs.02898

[B104] Harris KP Tepass U Cdc42 and vesicle trafficking in polarized cells Traffic 2010 11 10 1272 1279 10.1111/j.1600-0854.2010.01102.x 20633244

[B105] Wodarz A Molecular control of cell polarity and asymmetric cell division in Drosophila neuroblasts Curr Opin Cell Biol 2005 17 5 475 481 10.1016/j.ceb.2005.08.005 16099639

[B106] Goldstein B Macara IG The PAR proteins: fundamental players in animal cell polarization Dev Cell 2007 13 5 609 622 10.1016/j.devcel.2007.10.007 17981131 PMC2964935

[B107] St Johnston D Ahringer J Cell polarity in eggs and epithelia: parallels and diversity Cell 2010 141 5 757 774 10.1016/j.cell.2010.05.011 20510924

[B108] Balklava Z Pant S Fares H Grant BD Genome-wide analysis identifies a general requirement for polarity proteins in endocytic traffic Nat Cell Biol 2007 9 9 1066 1073 10.1038/ncb1627 17704769

[B109] Wissler F Labouesse M PARtners for endocytosis Nat Cell Biol 2007 9 9 1027 1029 10.1038/ncb0907-1027 17762895

[B110] Liu XF Tari PK Haas K PKM zeta restricts dendritic arbor growth by filopodial and branch stabilization within the intact and awake developing brain J Neurosci 2009 29 39 12229 12235 10.1523/JNEUROSCI.2842-09.2009 19793981 PMC6666143

[B111] Westmark PR Westmark CJ Wang S Levenson J O'Riordan KJ Burger C Malter JS Pin1 and PKMzeta sequentially control dendritic protein synthesis Sci Signal 2010 3 112 ra18 10.1126/scisignal.2000451 20215645 PMC2972507

[B112] Xue YX Luo YX Wu P Shi HS Xue LF Chen C Zhu WL Ding ZB Bao YP Shi J A memory retrieval-extinction procedure to prevent drug craving and relapse Science 2012 336 6078 241 245 10.1126/science.1215070 22499948 PMC3695463

[B113] Moncada D Viola H PKMzeta inactivation induces spatial familiarity Learn Mem 2008 15 11 810 814 10.1101/lm.1139508 18984560

[B114] Hrabetova S Sacktor TC Bidirectional regulation of protein kinase Mζ in the maintenance of long-term potentiation and long-term depression J Neurosci 1996 16 17 5324 5333 8757245 10.1523/JNEUROSCI.16-17-05324.1996 PMC6578881

[B115] Hrabetova S Sacktor TC Transient translocation of conventional protein kinase C isoforms and persistent downregulation of atypical protein kinase Mζ in long-term depression Brain Res Mol Brain Res 2001 95 1–2 146 152 11687286 10.1016/s0169-328x(01)00185-1

